# A national survey of lung cancer specialists’ views on low-dose CT screening for lung cancer in Korea

**DOI:** 10.1371/journal.pone.0192626

**Published:** 2018-02-08

**Authors:** Dong Wook Shin, Sohyun Chun, Young Il Kim, Seung Joon Kim, Jung Soo Kim, SeMin Chong, Young Sik Park, Sang-Yun Song, Jin Han Lee, Hee Kyung Ahn, Eun Young Kim, Sei Hoon Yang, Myoung Kyu Lee, Deog Gon Cho, Tae Won Jang, Ji Woong Son, Jeong-Seon Ryu, Moon-June Cho

**Affiliations:** 1 Department of Family Medicine & Supportive Care Center, Samsung Medical Center, Department of digital health, SAIHST, Sungkyunkwan University, Seoul, Korea; 2 International Clinic, Samsung Medical Center, Seoul, Korea; 3 Department of Radiation Oncology, Chungnam National University School of Medicine, Daejeon, Korea; 4 Department of Internal Medicine, Seoul St. Mary Hospital, The Catholic University of Korea College of Medicine, Seoul, Korea; 5 Department of Internal Medicine, lnha University Hospital, Inha University College of Medicine, Incheon, Korea; 6 Department of Radiology, Chung-Ang University Medical Center, Chung-Ang University College of Medicine, Seoul, Korea; 7 Department of Internal Medicine, Seoul National University Hospital, Seoul, Korea; 8 Department of Thoracic and Cardiovascular Surgery, Chonnam National University Hwasun Hospital, Chonnam National University Medical School, Hwasun, Korea; 9 Medical Correspondent & Social Policy Desk, Donga-A Ilbo, Seoul, Korea; 10 Department of Internal Medicine, Gachon University Gil Medical Center, Incheon, Korea; 11 Department of Internal Medicine, Gachon University Gil Medical Center, Incheon, Korea; 12 Department of Internal Medicine, Wonkwang University College of Medicine, Iksan, Korea; 13 Department of Internal Medicine, Wonju College of Medicine, Yonsei University, Wonju, Korea; 14 Department of Thoracic and Cardiovascular Surgery, St. Vincent's Hospital, College of Medicine, The Catholic University of Korea, Suwon, Korea; 15 Department of Internal Medicine, Kosin University Medical College, Pusan, Korea; 16 Department of Internal Medicine, Konyang University Hospital, Daejeon, Korea; University of Groningen, University Medical Center Groningen, NETHERLANDS

## Abstract

Lung cancer specialists play an important role in designing and implementing lung cancer screening. We aimed to describe their 1) attitudes toward low-dose lung computed tomography (LDCT) screening, 2) current practices and experiences of LDCT screening and 3) attitudes and opinions towards national lung cancer screening program (NLCSP). We conducted a national web-based survey of pulmonologists, thoracic surgeons, medical oncologists, and radiological oncologists who are members of Korean Association for Lung Cancer (N = 183). Almost all respondents agreed that LDCT screening increases early detection (100%), improves survival (95.1%), and gives a good smoking cessation counseling opportunity (88.6%). Most were concerned about its high false positive results (79.8%) and the subsequent negative effects. Less than half were concerned about radiation hazard (37.2%). Overall, most (89.1%) believed that the benefits outweigh the risks and harms. Most (79.2%) stated that they proactively recommend LDCT screening to those who are eligible for the current guidelines, but the screening propensity varied considerably. The majority (77.6%) agreed with the idea of NLCSP and its beneficial effect, but had concerns about the quality control of CT devices (74.9%), quality assurance of radiologic interpretation (63.3%), poor access to LDCT (56.3%), and difficulties in selecting eligible population using self-report history (66.7%). Most (79.2%) thought that program need to be funded by a specialized fund rather than by the National Health Insurance. The opinions on the level of copayment for screening varied. Our findings would be an important source for health policy decision when considering for NLCSP in Korea.

## Introduction

Lung cancer is one of the most common cancer and the leading cause of cancer deaths in Korea, Asia, and worldwide [1]. In Korea, 24,027 new lung cancers were diagnosed in 2014, and 17,440 died from lung cancer, accounting for 22.8% of all cancer deaths [2]. Prognosis of lung cancer remains poor, as it is often diagnosed at an advanced stage when curative treatment is no longer possible. In Korea, only 20% are diagnosed at a localized stage, and the 5-year survival rate of all lung cancer patients is only 21.9% [2]. Therefore, effective strategies have been sought to address this important public health problem.

The National Lung Screening Trial (NLST) demonstrated 20% and 6.7% relative reductions in lung cancer and all-cause mortality with annual low-dose computed tomography (LDCT) screening [3]. This result in 2011 has prompted multiple organizations to announce guidelines for lung cancer screening, including the American Cancer Society (ACS) [4], the American Society of Clinical Oncology (ASCO), the American Association of Thoracic Surgery (AATS) [5], the American College of Chest Physicians (ACCP)[6], and the U.S. Preventive Services Task Force [7]. A simulation study conducted in other settings, such as in China, also produced favorable results in terms of mortality outcomes [8].

However, there is still debate on the benefits and potential harms of lung cancer screening. Interim analyses of smaller studies showed no reduction in lung cancer mortality with LDCT screening [9]. In addition, LDCT screening carries a risk of negative effects such as false-positive results (i.e. 90% of the nodules discovered, found in 20% of patients according to the NLST definition and results), which increased the likelihood of receiving invasive diagnostic procedures and repeated irradiation. Therefore, despite the recommendations of specialist societies, the American Academy of Family Physician (AAFP) concluded that the evidence is insufficient to recommend for or against LDCT screening based on age and smoking history [10].

Since the Affordable Care Act that mandates private insurers to cover preventive services with a grade A and B by the USPSTF, in the US, Medicare and private insurers have initiated LDCT screening coverage, and LDCT is now widely adopted for lung cancer screening [11,12]. In Korea, the national screening guideline was revised to include LDCT screening starting September 2015, which happens to coincide with our study period. Demonstration project for National Lung Cancer Screening Program (NLCSP) is planned from 2017.

Healthcare providers play an important role in the informed decision about cancer screening by giving guidance to the patients and their family members about the benefits, potential harms, process, and the cost of LDCT screening. Lung cancer specialists in particular, are most likely to play an important role in designing and implementing lung cancer screening programs, since most primary clinics do not have the resources to implement LDCT. Furthermore, the opinion of these experts is the key in forming the national strategy for lung cancer prevention. Therefore, we aimed to describe their 1) attitudes regarding LDCT screening, 2) current practices and experiences of LDCT screening and 3) attitudes and opinions towards NLCSP, using a nationally representative sample of lung cancer specialists. In this particular study, we did not seek the opinion of primary care physicians.

## Methods

### Study design and subjects

The present study was conducted as a part of a national web-based survey to explore the views of lung cancer specialists regarding the smoking-related policies in October 2015. The institutional review board of the Inha University Hospital, Incheon, Korea approved this study (IRB no. 15–053).

Among members of Korean Association for Lung Cancer, we included pulmonologists, thoracic surgeons, medical oncologists, and radiological oncologist. We limited our participants to lung cancer specialist physicians who provide direct care to lung cancer patients and their family, as some of our question items require a certain level of clinical interaction experience (e.g. current practice, physician-perceived reasons for refusal of lung cancer screening). Therefore, thoracic radiologists or pathologists were not included in our study. A total of 383 physicians were identified as eligible subjects, who received up to 3 invitations to participate in the survey with text message reminders. For non-responders, a single reminder phone call was made to encourage participation.

Of 383 eligible subjects, 196 agreed to participate in the study (51.3% participation rate). However, 13 of them did not provide sufficient responses to the LDCT screening questionnaire and were therefore excluded from the analysis, leading to a final sample of 183 in the current study (47.8% effective response rate). Details of survey design are described elsewhere [13].

#### Survey instrument and administration

Given the absence of a validated questionnaire which assesses physicians’ attitudes toward LDCT screening, we developed a questionnaire based on previous literature [12,14]. The questionnaire appraised attitudes regarding LDCT screening [14], current practices and experiences of the specialists [14], and their attitudes and opinions towards NLCSP [14]. In addition to the questionnaire, the survey consisted of items measuring personal and professional demographics such as age, sex, specialty, years from board certification, workplace (university hospital vs. specialized cancer center, private vs. public, and geographic location), and patient volume (clinical practice sessions per week, average number of overall patients and lung cancer outpatients per week). The survey was reviewed and piloted for content and clarity by a committee, of which member includes 12 lung cancer specialists and a public health researcher who is an expert in survey methodology.

#### Attitudes towards lung cancer screening with LDCT

Physicians were asked whether they agree or disagree with the statements regarding benefits, potential harms, and cost of lung cancer screening with LDCT on a 4-point Likert scale (strongly disagree to strongly agree). Questions regarding benefits included early detection of lung cancer, increased survival [10,14], smoking cessation counseling opportunities and actual resultant in smoking cessation [15]. Questions regarding potential harms included medical and psychological consequences of false positive results [12,14,16,17], false reassurance and continued smoking from a negative result [2,12], and radiation hazards [12,16]. Cost burden was also asked [11,14]. Following these questions, respondents were asked to assess the net benefit of LDCT screening, considering the potential benefits, risks, and cost.

#### Current lung cancer screening practices

Three clinical vignettes were used to assess current lung cancer screening practices. Physicians were asked if they would offer LDCT screening to 1) an asymptomatic long-term smoker who meets the current eligibility criteria of the guideline (e.g. ≥30 pack-year and 55–79 years old) [5,7,18], 2) an asymptomatic long-term smoker who does not meet the eligibility criteria (e.g. 20 to 30 pack-year and 40–50 years old), 3) a son of lung cancer patient who is not eligible (e.g. 10 pack-year of smoking history and has worries about lung cancer). Response options were “proactively recommend”, “recommend when the patient seeks for an expert opinion”, and “not recommend”. Responses to clinical vignettes have been shown to correlate with clinical behavior, and are also known to be a useful tool for assessing clinical decision-making and guideline-based practice [19,20].

Regarding reimbursement and payment issue [14,16], we asked how they have their patients get LDCT for screening. The response options were “through insurance coverage”, “out of pocket expenses”, “through a referral to a health screening center”, and “not recommend LDCT for screening purposes”.

Physicians were also asked about their experiences regarding the patients’ refusal to take LDCT for screening upon offer. Six plausible items were provided as likely reasons for patients’ reluctance to LDCT screening, based on cancer screening literature and clinical experiences [11,12,21]: “lack of knowledge on lung cancer risks [12,16]”, “denial of their own risk for lung cancer [22]”, “fear of actual lung cancer detection [12,21]”, “lack of perceived benefit of early lung cancer detection [12,21]”, “concern about the screening cost [12,16,22]”, and “conflict of interest that doctor recommended the screening for the doctor’s own financial benefit [23]”.

#### Attitudes and opinions toward NLCSP

Physicians were also asked whether they think LDCT should be part of the National Cancer Screening Program (NCSP). Expected benefits of having LDCT screening in the NCSP were asked in terms of effectiveness, cost-effectiveness, and equity [12,17]. Questions for perceived barriers in the implementation of LDCT screening as a NCSP included unavailability of LDCT in primary care [12,17], quality control of the equipment and personnel perspectives [16], selection of indicated persons, and potential forfeit. Physicians were to answer each item on a 4-point Likert scale (strongly disagree to strongly agree). Opinions on public funding for NLCSP were obtained by asking them whether indicated patients should bear all or only part of the cost for screening, whether the program should be funded and how.

#### Statistical analysis

Descriptive statistics were used to calculate responses provided to the questions. All statistical analyses were performed using STATA version 14.0 (STATA corp, College Station, TX, USA).

## Results

### Respondent characteristics

Most participants were male (82.0%) and practiced in a university hospital or a hospital with cancer center (88.0%) **(Table 1).** Respondents were comprised of pulmonologists (59.0%), thoracic surgeons (23.0%), medical oncologists (10.4%), and radiation oncologists (7.7%). The average age was 44.4 years (SD 7.2 years), and mean year from board certification was 13.6 years (SD 7.2 years).

**Table 1:** Characteristics of the participants (N = 183).

	N or mean	% or SD
Age (mean, SD)	44.4	7.2
Gender		
Male	150	82.0
Female	33	18.0
Specialty		
Pulmonologist	108	59.0
Thoracic surgeon	42	23.0
Medical oncologist	19	10.4
Radiation oncologist	14	7.7
Years from board certification (mean, SD)	13.6	7.2
Hospital type		
University hospital	149	81.4
Cancer specialty hospital	12	6.6
Secondary hospital	13	7.1
Non-response	9	4.9
Hospital type		
Public hospital	46	25.1
Private hospital	128	70.0
Non-response	9	4.9
Number of clinical sessions per week (mean, SD)	4.1	1.4
Number of lung cancer patients per week (mean, SD)	32.1	40.4

SD: standard deviation

### Attitudes toward LDCT screening

Almost all physicians agreed that LDCT screening increases early detection (100%) and survival (95.1%), and most thought that it gives a good opportunity to counsel smoking cessation (88.6%), and that it can increase the chances of smoking cessation in their patients (61.2%). The majority of physicians believed that the risk of false positive is too high (79.8%), and that false positive results can produce psychological distress (88.0%) and physical harms (55.2%). Only a minority of physicians believed that false positive results would incur unnecessary tests (16.9%).

More than half of the physicians were concerned that negative results can give false reassurance (59.1%) or allow smokers to continue smoking (63.4%). Less than half believed that radiation hazard is clinically meaningful (37.2%), and that the cost of screening is burdensome for people with an average income (40.5%). Overall, the majority of physicians (89.1%) agreed that the LDCT screening benefits outweigh the potential risk and harms **(Table 2).**

**Table 2:** Attitudes toward lung cancer screening by low-dose computed tomography among lung cancer specialist physicians.

	Strongly disagree		Disagree		Agree		Strongly agree	
	N	%	N	%	N	%	N	%
Potential benefits and effectiveness								
Increases early detection	0	0.0	0	0.0	105	57.4	78	42.6
Improves survival by early detection	1	0.6	8	4.4	124	67.8	50	27.3
Provides opportunity for smoking cessation counseling	1	0.6	20	10.9	124	67.8	38	20.8
Increases smoking cessation	4	2.2	67	36.6	94	51.4	18	9.8
Potential harms								
Risk for false positive result is too high.	1	0.6	36	19.7	114	62.3	32	17.5
False positive result incurs unnecessary further tests.	36	19.7	116	63.4	31	16.9	0	0.0
False positive result produces psychological distress.	1	0.6	21	11.5	121	66.1	40	21.9
False positive result produces physical harms.	4	2.2	78	42.6	87	47.5	14	7.7
Negative result gives false reassurance.	2	1.1	74	40.4	94	51.4	13	7.1
Negative result lead smokers to continue smoking.	4	2.2	63	34.4	107	58.5	9	4.9
Radiation hazard is clinically meaningful.	16	8.7	99	54.1	67	36.6	1	0.6
Cost								
Cost is burdensome to people with an average income.	5	2.7	104	56.8	66	36.1	8	4.4
Overall evaluation								
Taken all, benefits outweigh the risks.	1	0.6	19	10.4	124	67.8	39	21.3

### Current practice and experience of LDCT screening

In response to the patient vignettes, most physicians (79.2%) indicated that they would proactively recommend LDCT screening to those who are eligible according to the current guidelines, while others (19.7%) would recommend it when the patients seek for an expert opinion. For a long-term smoker who does not meet the current eligible criteria, physicians stated that they would recommend screening proactively (68.3%) or upon the patient’s request for an expert opinion (24.0%). For a smoker whose parent(s) had been diagnosed with lung cancer, has a 10 pack-year smoking history of his own, and worries about getting lung cancer, only 9.3% of the physicians answered that they would proactively recommend screening. Most others (76.0%) would recommend it upon request for their opinion, while 14.8% would discourage screening **(Table 3)**.

**Table 3:** Current practice of lung cancer screening recommendation among lung cancer specialist physicians.

	Proactively recommend		Recommend when the patient seeks opinion		Do not recommend	
Clinical scenarios	N	%	N	%	N	%
Smoking history ≥30 pack-year & age 55 to 79 years (indicated for lung cancer screening by current guidelines)	145	79.2	36	19.7	2	1.1
Smoking history 20 to 30 pack-year & age 40 to 59 years (not indicated for lung cancer screening by current guidelines)	44	24.0	125	68.3	14	7.7
Smoking history 10 pack-year, son of lung cancer patient and worries about lung cancer	17	9.3	139	76.0	27	14.8

When asked about ordering LDCT for the screening eligible population, half (50.3%) of physicians reported that they order it under insurance (30% copayment); approximately a quarter (27.3%) order it as out-of-pocket expenses and less than a quarter (19.1%) refer patients to health screening centers where patients get private screening at their own cost.

Physician-perceived reasons of rejecting LDCT screening among patients who are currently indicated included concerns about cost (73.8%), denial of their own risk (67.2%), lack of perceived benefit (54.1%), fear of actual lung cancer detection (53.5%), suspicion for physicians’ own benefit (48.6%), and lack of knowledge of lung cancer risk (41.6%) **(Table 4)**.

**Table 4:** Physician-perceived reasons for refusal of lung cancer screening among people who are indicated.

	Frequently		Often		Rarely		Never	
	N	%	N	%	N	%	N	%
Lack of knowledge of lung cancer risks (e.g. does not know that smoking increases lung cancer risk)	8	4.4	68	37.2	86	47.0	21	11.5
Denial of their own lung cancer risk (e.g. knows that smoking increases lung cancer risk, but think he/she will be OK)	16	8.7	107	58.5	52	28.4	8	4.4
Fear of actual lung cancer detection (e.g. fear that lung cancer will be detected by screening)	7	3.8	91	49.7	73	39.9	12	6.6
Lack of perceived benefit of early lung cancer detection (e.g. thinks that he/she will die anyway once he/she gets lung cancer)	15	8.2	84	45.9	72	39.3	12	6.6
Concern about the cost	43	23.5	92	50.3	44	24.0	4	2.2
Suspicion that doctors recommend screening for their own good	13	7.1	76	41.5	80	43.7	14	7.7

### Attitudes towards and opinions on NLCSP

Most physicians (77.6%) agreed with the idea of national lung cancer screening program (NLCSP). Most respondents strongly agreed or agreed that NLCSP would reduce morality (87.4%), will be cost-effective (83.6%) and cost-saving (68.4%), and that it would improve health equity (77.1%). However, at the same time, majority were concerned about the quality control of CT devices (74.9%), quality assurance of radiological interpretation (63.3%), poor access to LDCT in primary care (56.3%). They also believed that there may be difficulties in selecting eligible screening population because they would have to rely on self-reports on the smoking history (66.7%), and that people may fabricate their smoking history just to become eligible for screening (83.6%) **(Table 5).**

**Table 5:** Attitudes towards national lung cancer screening program (NLCSP) among lung cancer specialist physicians.

	Strongly disagree		Disagree		Agree		Strongly agree	
	N	%	N	%	N	%	N	%
Expected benefit								
NLCSP will reduce mortality from lung cancer.	0	0.0	21	11.5	119	65.0	41	22.4
NLCSP will be a cost-effective program.	0	0.0	28	15.3	119	65.0	34	18.6
NLCSP will be cost-saving.	1	0.6	55	30.1	96	52.5	29	15.9
As smoking is disproportionally prevalent in low income bracket, providing NLCSP would be beneficial to reduce health inequality.	2	1.1	38	20.8	124	67.8	17	9.3
Potential Barriers								
Access to LDCT will be not good as primary care facilities do not have the resources.	3	1.6	75	41.0	92	50.3	11	6.0
Quality control will be an issue as the quality of CT device varies in each facility.	3	1.6	41	22.4	112	61.2	25	13.7
Quality assurance will be not easy as the quality of radiologic interpretation will vary among radiologists.	2	1.1	45	24.6	109	59.6	25	13.7
Selecting indicated patients will be not easy as the smoking history (duration & amount) is obtained self-reportedly.	3	1.6	56	30.6	110	60.1	12	6.6
People may fabricate their smoking history to get lung cancer screening if NLCSP become available.	1	0.6	27	14.8	129	70.5	24	13.1

Regarding self-pay proportion of the screening cost, most physicians (77.6%) endorsed some level of copayment, with most frequently answered proportion to be 50%. Minority physicians advocated no copayment at all (12.6%), and even less (7.7%) insisted on 100% out-of-pocket screening for the eligible population. Majority (79.2%) agreed that the program would need to be supported by a specialized fund, such as the Health Promotion Fund from tobacco taxes, rather than by the National Health Insurance **(Table 6)**.

**Table 6:** Opinions on the public funding of national lung cancer screening program among lung cancer specialist physicians.

	N	%
Appropriate amount of out-of-pocket payment for the NLCSP		
Free of charge	23	12.6
Copayment (appropriate level of copayment: ________ %)	146	77.6
5%	6	3.3
10%	23	12.6
15%	1	0.6
20%	23	12.6
25%	1	0.6
30%	23	12.6
40%	2	1.1
45%	2	1.1
50%	60	32.8
60%	1	0.6
Missing	4	2.2
100% out-of-pocket	14	7.7
Non-response	4	2.2
Appropriate public funding methods for the NLCSP		
General budget (national, regional0	3	1.6
National Health insurance (insurance premium)	22	12.0
Specialized fund (e.g., Health promotion fund from tobacco tax)	145	79.2
Out-of-pocket cost	11	6.0
Non-response	2	1.1

CT: computed tomography

## Discussion

To our knowledge, this is the first results of a national survey of lung cancer specialist physicians’ attitudes toward and current practice of LDCT screening for lung cancer. Timely investigation of an emerging health issue, use of a nationwide sample covering all geographic areas, specialty and types of hospital are unique strengths of this study.

### Attitudes towards lung cancer screening by low-dose CT

Physician knowledge and attitude towards LDCT screening is important as it can determine their recommendation, which is critical for patient’s uptake or actual ordering of LDCT screening [11,12,14]. Overall, nearly 90% of our study participants agreed that benefits of LDCT screening for guideline-eligible population outweigh the potential risks, showing their generally positive attitudes.

Our study participants strongly believed that LDCT is effective in early detection of cancer and improving survival. In fact, recent evidence showed that lung cancer screening with LDCT is more effective than breast or colorectal cancer screening (number needed to treat 320 vs. 1339 and 871, respectively) [12]. Lung cancer specialists seem to acknowledge such benefits, contrary to primary care physicians who were generally skeptical about LDCT screening [16].

According to NLST data, around 30% of people who underwent LDCT screening had at least one false positive result, and 0.3% will have a major complication from a following invasive diagnostic procedure [18]. While lung cancer specialists generally acknowledged the high risk of false positive results and the possible subsequent psychological and physical harms, they did not think it incurred further unnecessary tests. It can be interpreted that even though some patients may experience negative consequences from false positive results, lung cancer specialists generally think that follow-up or further diagnostic tests is still worthwhile and not ‘unnecessary’. This is also in contrast with the views of primary care physicians who are often concerned about false-positive results and their downstream consequences, such as further diagnostic testing and invasive procedures, psychological stress, missing work, and financial issues as well [16]. More recently published NELSON or UKLS trials have used a different definition of false positive: requirement for referral to the pulmonologist and further diagnostic investigation (around 3.5%) without subsequent diagnosis of lung cancer [24,25], unlike NLST, in which every individual who had an additional CT scan before a repeat annual screen was considered positive. This such interpretation of the term false-positive may potentially attenuate the perceived harms coming from false-positive results. Furthermore, the advent of volumetric analysis of detected nodules would be able to actually reduce the harms related to false positive results, relieving the physicians’ concerns [25,26].

Integrated smoking cessation services to lung cancer screening was emphasized to take the opportunity of ‘teachable moment’ and improve cost-effectiveness of the LDCT screening program [15,22,27]. While most agreed that discussing LDCT screening provides a good opportunity for smoking cessation counseling, some of them were skeptical about whether it actually improves smoking cessation. This might be linked to the perceived-harm of false negative result; more than half of the participants agreed that patients can be falsely reassured about their health and continue smoking. Currently, evidence is scarce and contradictory whether a negative screening test will decrease risk perception or provide false reassurance, and whether LDCT screening, either by itself or combined with smoking cessation services, has a positive effect on smoking behaviors [15,28]. Further research will be needed to find out an optimal method of integrating smoking cessation services in the upcoming NLCSPs.

Around two-thirds disagreed that radiation hazard is clinically meaningful, and this is concordant with the recent analyses that radiation exposure and cancer risk from LDCT screening is acceptable, even if non-negligible, considering the substantial effectiveness of mortality reduction [29]. Regarding cost, approximately 40% agreed that cost for LDCT screening is burdensome to people with an average salary.

### Current practice

Virtually all lung cancer specialists answered that they recommend LDCT screening to the guideline-eligible patient, suggesting that they are familiar with the current lung cancer screening guidelines. While 20% did so only when patients seek expert opinions, this might be because there is currently no established lung cancer screening program in Korea and the cost is generally paid out-of-pocket. This is contrary to the US primary care providers who were more cautious and ambivalent about offering LDCT screening, preferring to take a “wait and see” approach [16].

Vast majority also answered that they would recommend LDCT screening to the long-term smoker who did not meet the current criteria, and to the son of lung cancer patient, either proactively or when asked for their opinions. This largely reflects the positive attitudes of our study participants toward LDCT screening in terms of effectiveness and cost-effectiveness. This is also closely related to the fee-for-service healthcare system in Korea where patients’ request is an important motive for preventive services [14]. Several guidelines have expanded the eligibility criteria to include individuals who are ≥50 years and have ≥ 20 pack-year smoking history or have an additional risk factor such as lung cancer family history [5,30]. However, such practice could raise the issue of over-screening. Extrapolation of the current recommendations to less high risk groups should be done with extreme caution, as its effectiveness and cost-effectiveness would be lower than in the higher risk group that is currently guideline-eligible. It would incur substantial cost implications as well, not only from the screening itself but also from consequent tests and procedures after a positive screening result [31,32]. Additional effort is warranted to establish effectiveness and cost-effectiveness results pertinent to Korea, to facilitate a shared decision making on lung cancer screening.

Without established national screening program or reimbursement guidelines, there was much variation in the prescription pattern. Half of our study participants prescribed LDCT under insurance (probably with some diagnostic code for reimbursement) and the other half 100% out-of-pocket. There is a need for reasonable criteria to reduce the variation.

Lack of knowledge of screening benefit, risk denial and cost burden are the common barriers of screening practice; it should be addressed in the national policy to implement and disseminate LDCT screening.

### Attitudes and opinions towards NLCSP

In the US, screening for lung cancer with LDCT is estimated to cost $81,000 per quality-adjusted life year gained [32], and implementation of LDCT screening program on a nationwide scale was estimated to cost $1.3~2.0 billion annually [33]. To our knowledge, there has not been a formal estimation in Korea on LDCT screening. However, lung cancer specialists, consistent with their attitudes toward LDCT screening itself, generally had a positive view on the cost-effectiveness of the NLCSP. This is in contrast to that of US primary care physicians, who had ethical concerns about allocating limited healthcare resources to LDCT screening rather than to other tobacco control programs, or to older, heavy smokers rather than young population who are likely to result in greater social benefit [16].

Our respondents generally agreed on the positive impact on health equity, as smoking is more prevalent in the lower social class. Previous studies have shown discrepant views on implementation of NLCSP in terms of health equity: some argued that it would improve equity as underprivileged people are often excluded from latest advances, while others thought it could burden patients who cannot afford follow-up care [16]. Further study is warranted to provide empirical data on the impact of NLCSP on health equity issues.

Respondents had concerns about infrastructure for NLCSP, such as proper access to the program and assuring high quality screening in terms of equipment and personnel. A US study found that many primary care physicians were not sure about whether LDCT is available near their practice and whether it is covered by Medicare/Medicaid, and therefore did not refer their high risk patients for screening [17]. High quality of device and accurate radiologic interpretation are essential to ensure screening benefit and to reduce potential harms. Current guidelines recommend that lung cancer screening should only be performed according to a standard protocol at centers that are able to guarantee rigorous quality control, and should be linked to multidisciplinary management team with well-developed program of minimally invasive thoracic surgery [26,34,35]. Therefore, quality control plan should be an important consideration in the implementation and dissemination of the NLCSP. Financing the NLCSP is another important issue in the implementation of NLCSP. Cost burden was the most frequently cited barrier to LDCT screening in our study, which is consistent with the US findings that few patients are willing to pay out of pocket [12]. Respondents had varying opinions on the level of copayment. While most agreed on the need for copayment, there was no universal agreement on the proportion of copayment. Others either advocated free screening or insisted 100% out-of-pocket service. However, when it came to public funding for the NLCSP, most advocated using a specialized fund from tobacco taxes. This might be due to a societal concern that non-smokers would be paying premiums for smokers to receive screening, which was mentioned in a previous US study [16].

### Limitations

One notable limitation of our study is that we surveyed lung cancer specialists only. Thoracic radiologists or pathologists were not included. Most guideline-eligible long-term smokers do not come in direct contact with lung cancer specialists, but are more likely meet with primary care physicians for healthcare including health checkup or care of chronic illnesses, such as hypertension or diabetes. Future study is warranted to investigate the views of primary care physicians in Korea. However, the attitudes of lung cancer specialists are particularly important as they can influence the clinical practice guidelines, and primary care physicians adopt and implement such guidelines in their practice [16]. Other potential limitation is a relatively small sample size and a low response rate, which can create nonresponse bias. In addition, although the survey items were developed mainly by adopting items used in previous studies, the questionnaire was neither guided by a theoretical framework nor validated psychometrically. Finally, our study findings need to be interpreted in the Korean context, and the generalizability to other countries are limited because of the differences in culture and health care system. However, the lung cancer specialists’ view on the potential benefit, harm, and barriers of lung cancer screening identified from this study would help the policy makers from other countries when planning their own program.

## Conclusions

Our results demonstrated that most Korean lung cancer specialists are positive about LDCT screening, recommend LDCT screening concordant with the current guideline, but also find difficulties with patients’ refusal for various reasons. They are also generally positive about the national lung cancer screening program, but have concerns about access, quality control, and objective selection of the eligible patients using self-reported smoking history. Decision for implementing NLCSP should be made in the broader context of overall benefits, risks, and cost for screening program. Healthcare system should carefully plan and allocate sufficient resources to ensure success of an organized screening program. Our findings from this study on the attitudes of lung cancer specialists on NLCSP would be an important source for future health policy decision.

## References

[1] Siegel RL, Miller KD, Jemal A. Cancer statistics, 2016. CA Cancer J Clin. 2016;66(1):7–30. doi: 10.3322/caac.21332 . 26742998 10.3322/caac.21332

[2] Jung KW, Won YJ, Oh CM, Kong HJ, Lee DH, Lee KH, et al Cancer Statistics in Korea: Incidence, Mortality, Survival, and Prevalence in 2014. Cancer research and treatment: official journal of Korean Cancer Association. 2017;49(2):292–305. doi: 10.4143/crt.2017.118 ; PubMed Central PMCID: PMC5398380. 28279062 10.4143/crt.2017.118 PMC5398380

[3] National Lung Screening Trial Research T, Aberle DR, Adams AM, Berg CD, Black WC, Clapp JD, et al Reduced lung-cancer mortality with low-dose computed tomographic screening. The New England journal of medicine. 2011;365(5):395–409. doi: 10.1056/NEJMoa1102873 ; PubMed Central PMCID: PMC4356534. 21714641 10.1056/NEJMoa1102873 PMC4356534

[4] Wender R, Fontham ET, Barrera E Jr., Colditz GA, Church TR, Ettinger DS, et al American Cancer Society lung cancer screening guidelines. CA Cancer J Clin. 2013;63(2):107–17. doi: 10.3322/caac.21172 ; PubMed Central PMCID: PMCPMC3632634. 23315954 10.3322/caac.21172 PMC3632634

[5] Jaklitsch MT, Jacobson FL, Austin JH, Field JK, Jett JR, Keshavjee S, et al The American Association for Thoracic Surgery guidelines for lung cancer screening using low-dose computed tomography scans for lung cancer survivors and other high-risk groups. J Thorac Cardiovasc Surg. 2012;144(1):33–8. doi: 10.1016/j.jtcvs.2012.05.060 . 22710039 10.1016/j.jtcvs.2012.05.060

[6] Detterbeck FC, Mazzone PJ, Naidich DP, Bach PB. Screening for lung cancer: Diagnosis and management of lung cancer, 3rd ed: American College of Chest Physicians evidence-based clinical practice guidelines. Chest. 2013;143(5 Suppl):e78S–92S. doi: 10.1378/chest.12-2350 ; PubMed Central PMCID: PMCPMC3749713. 23649455 10.1378/chest.12-2350 PMC3749713

[7] Moyer V. Lung cancer prevention and screening. Oncology. 2014;28(5):449–50. . 25004663

[8] Wang Z, Han W, Zhang W, Xue F, Wang Y, Hu Y, et al Mortality outcomes of low-dose computed tomography screening for lung cancer in urban China: a decision analysis and implications for practice. Chinese journal of cancer. 2017;36(1):57 doi: 10.1186/s40880-017-0221-8 . 28709441 10.1186/s40880-017-0221-8 PMC5512753

[9] Infante M, Cavuto S, Lutman FR, Passera E, Chiarenza M, Chiesa G, et al Long-Term Follow-up Results of the DANTE Trial, a Randomized Study of Lung Cancer Screening with Spiral Computed Tomography. American journal of respiratory and critical care medicine. 2015;191(10):1166–75. doi: 10.1164/rccm.201408-1475OC . 25760561 10.1164/rccm.201408-1475OC

[10] American Academy of Family Physicians. Clinical Preventive Service Recommendation. Lung cancer 2013 [cited 2017 May 13]. Available from: http://www.aafp.org/patient-care/clinical-recommendations/all/lungcancer.html.

[11] Iaccarino JM, Clark J, Bolton R, Kinsinger L, Kelley M, Slatore CG, et al A National Survey of Pulmonologists' Views on Low-Dose Computed Tomography Screening for Lung Cancer. Annals of the American Thoracic Society. 2015;12(11):1667–75. doi: 10.1513/AnnalsATS.201507-467OC . 26368003 10.1513/AnnalsATS.201507-467OC

[12] Lewis JA, Petty WJ, Tooze JA, Miller DP, Chiles C, Miller AA, et al Low-Dose CT Lung Cancer Screening Practices and Attitudes among Primary Care Providers at an Academic Medical Center. Cancer epidemiology, biomarkers & prevention: a publication of the American Association for Cancer Research, cosponsored by the American Society of Preventive Oncology. 2015;24(4):664–70. doi: 10.1158/1055-9965.EPI-14-1241 ; PubMed Central PMCID: PMC4383689. 25613118 10.1158/1055-9965.EPI-14-1241 PMC4383689

[13] Shin DW, Kim YI, Kim SJ, Kim JS, Chong S, Park YS, et al Lung cancer specialist physicians' attitudes towards e-cigarettes: A nationwide survey. PloS one. 2017;12(2):e0172568 doi: 10.1371/journal.pone.0172568 ; PubMed Central PMCID: PMCPMC5325291. 28235068 10.1371/journal.pone.0172568 PMC5325291

[14] Henderson S, DeGroff A, Richards TB, Kish-Doto J, Soloe C, Heminger C, et al A qualitative analysis of lung cancer screening practices by primary care physicians. Journal of community health. 2011;36(6):949–56. doi: 10.1007/s10900-011-9394-2 . 21442338 10.1007/s10900-011-9394-2

[15] Fucito LM, Czabafy S, Hendricks PS, Kotsen C, Richardson D, Toll BA, et al Pairing smoking-cessation services with lung cancer screening: A clinical guideline from the Association for the Treatment of Tobacco Use and Dependence and the Society for Research on Nicotine and Tobacco. Cancer. 2016;122(8):1150–9. doi: 10.1002/cncr.29926 ; PubMed Central PMCID: PMC4828323. 26916412 10.1002/cncr.29926 PMC4828323

[16] Hoffman RM, Sussman AL, Getrich CM, Rhyne RL, Crowell RE, Taylor KL, et al Attitudes and Beliefs of Primary Care Providers in New Mexico About Lung Cancer Screening Using Low-Dose Computed Tomography. Preventing chronic disease. 2015;12:E108 doi: 10.5888/pcd12.150112 ; PubMed Central PMCID: PMC4509091. 26160294 10.5888/pcd12.150112 PMC4509091

[17] Ersek JL, Eberth JM, McDonnell KK, Strayer SM, Sercy E, Cartmell KB, et al Knowledge of, attitudes toward, and use of low-dose computed tomography for lung cancer screening among family physicians. Cancer. 2016;122(15):2324–31. doi: 10.1002/cncr.29944 . 27294476 10.1002/cncr.29944

[18] Heleno B, Rasmussen JF, Brodersen J. Reduced lung-cancer mortality with CT screening. The New England journal of medicine. 2011;365(21):2036; author reply 7–8. doi: 10.1056/NEJMc1110293#SA2 . 22111729 10.1056/NEJMc1110293

[19] Peabody JW, Luck J, Glassman P, Dresselhaus TR, Lee M. Comparison of vignettes, standardized patients, and chart abstraction: a prospective validation study of 3 methods for measuring quality. Jama. 2000;283(13):1715–22. . 10755498 10.1001/jama.283.13.1715

[20] Peabody JW, Luck J, Glassman P, Jain S, Hansen J, Spell M, et al Measuring the quality of physician practice by using clinical vignettes: a prospective validation study. Annals of internal medicine. 2004;141(10):771–80. . 15545677 10.7326/0003-4819-141-10-200411160-00008

[21] Quaife SL, McEwen A, Janes SM, Wardle J. Attitudes towards lung cancer screening within socioeconomically deprived and heavy smoking communities: a qualitative study. The Lancet. 384:S16 doi: 10.1016/S0140-6736(14)62142-5 10.1111/hex.12481 PMC5513004 27397651

[22] Kanodra NM, Pope C, Halbert CH, Silvestri GA, Rice LJ, Tanner NT. Primary Care Provider and Patient Perspectives on Lung Cancer Screening. A Qualitative Study. Annals of the American Thoracic Society. 2016;13(11):1977–82. doi: 10.1513/AnnalsATS.201604-286OC . 27676369 10.1513/AnnalsATS.201604-286OC

[23] Shin DW, Cho J, Yang HK, Kim SY, Park B, Cho B, et al Oncologists' Experience with Patients with Second Primary Cancer and the Attitudes toward Second Primary Cancer Screening: A Nationwide Survey. Cancer research and treatment: official journal of Korean Cancer Association. 2015;47(4):600–6. doi: 10.4143/crt.2014.162 ; PubMed Central PMCID: PMC4614218. 25687866 10.4143/crt.2014.162 PMC4614218

[24] Field JK, Duffy SW, Baldwin DR, Brain KE, Devaraj A, Eisen T, et al The UK Lung Cancer Screening Trial: a pilot randomised controlled trial of low-dose computed tomography screening for the early detection of lung cancer. Health technology assessment. 2016;20(40):1–146. doi: 10.3310/hta20400 ; PubMed Central PMCID: PMC4904185. 27224642 10.3310/hta20400 PMC4904185

[25] Horeweg N, van der Aalst CM, Vliegenthart R, Zhao Y, Xie X, Scholten ET, et al Volumetric computed tomography screening for lung cancer: three rounds of the NELSON trial. The European respiratory journal. 2013;42(6):1659–67. doi: 10.1183/09031936.00197712 . 23845716 10.1183/09031936.00197712

[26] Oudkerk M, Devaraj A, Vliegenthart R, Henzler T, Prosch H, Heussel CP, et al European position statement on lung cancer screening. The Lancet Oncology. 2017;18(12):e754–e66. doi: 10.1016/S1470-2045(17)30861-6 . 29208441 10.1016/S1470-2045(17)30861-6

[27] Villanti AC, Jiang Y, Abrams DB, Pyenson BS. A cost-utility analysis of lung cancer screening and the additional benefits of incorporating smoking cessation interventions. PloS one. 2013;8(8):e71379 doi: 10.1371/journal.pone.0071379 ; PubMed Central PMCID: PMC3737088. 23940744 10.1371/journal.pone.0071379 PMC3737088

[28] Park ER, Gareen IF, Jain A, Ostroff JS, Duan F, Sicks JD, et al Examining whether lung screening changes risk perceptions: National Lung Screening Trial participants at 1-year follow-up. Cancer. 2013;119(7):1306–13. doi: 10.1002/cncr.27925 ; PubMed Central PMCID: PMC3604047. 23280348 10.1002/cncr.27925 PMC3604047

[29] Rampinelli C, De Marco P, Origgi D, Maisonneuve P, Casiraghi M, Veronesi G, et al Exposure to low dose computed tomography for lung cancer screening and risk of cancer: secondary analysis of trial data and risk-benefit analysis. BMJ. 2017;356:j347 doi: 10.1136/bmj.j347 . 28179230 10.1136/bmj.j347 PMC5421449

[30] National Comprehensive Cancer Network. NCCN Clinical Practice Guidelines in Oncology: Lung Cancer Screening 2016 [cited 2017 May 20]. Available from: https://www.nccn.org/store/login/login.aspx?ReturnURL = https://www.nccn.org/professionals/physician_gls/pdf/lung_screening.pdf.

[31] Doria-Rose VP, White MC, Klabunde CN, Nadel MR, Richards TB, McNeel TS, et al Use of lung cancer screening tests in the United States: results from the 2010 National Health Interview Survey. Cancer epidemiology, biomarkers & prevention: a publication of the American Association for Cancer Research, cosponsored by the American Society of Preventive Oncology. 2012;21(7):1049–59. doi: 10.1158/1055-9965.EPI-12-0343 ; PubMed Central PMCID: PMC3392469. 22573798 10.1158/1055-9965.EPI-12-0343 PMC3392469

[32] Black WC, Gareen IF, Soneji SS, Sicks JD, Keeler EB, Aberle DR, et al Cost-effectiveness of CT screening in the National Lung Screening Trial. The New England journal of medicine. 2014;371(19):1793–802. doi: 10.1056/NEJMoa1312547 ; PubMed Central PMCID: PMC4335305. 25372087 10.1056/NEJMoa1312547 PMC4335305

[33] Goulart BH, Bensink ME, Mummy DG, Ramsey SD. Lung cancer screening with low-dose computed tomography: costs, national expenditures, and cost-effectiveness. Journal of the National Comprehensive Cancer Network: JNCCN. 2012;10(2):267–75. . 22308519 10.6004/jnccn.2012.0023

[34] Pedersen JH, Rzyman W, Veronesi G, D'Amico TA, Van Schil P, Molins L, et al Recommendations from the European Society of Thoracic Surgeons (ESTS) regarding computed tomography screening for lung cancer in Europe. European journal of cardio-thoracic surgery: official journal of the European Association for Cardio-thoracic Surgery. 2017;51(3):411–20. doi: 10.1093/ejcts/ezw418 . 28137752 10.1093/ejcts/ezw418 PMC6279064

[35] Postmus PE, Kerr KM, Oudkerk M, Senan S, Waller DA, Vansteenkiste J, et al Early and locally advanced non-small-cell lung cancer (NSCLC): ESMO Clinical Practice Guidelines for diagnosis, treatment and follow-up. Annals of oncology: official journal of the European Society for Medical Oncology. 2017;28(suppl_4):iv1–iv21. doi: 10.1093/annonc/mdx222 . 28881918 10.1093/annonc/mdx222

